# Large-Scale Multigenome-Wide Study Predicts the Existence of Transmembrane Phosphotransfer Proteins in Plant Multistep Phosphorelay Signaling Pathway

**DOI:** 10.3390/ijms27010240

**Published:** 2025-12-25

**Authors:** Sergey N. Lomin, Wolfram G. Brenner, Ekaterina M. Savelieva, Dmitry V. Arkhipov, Georgy A. Romanov

**Affiliations:** 1 Timiryazev Institute of Plant Physiology, Russian Academy of Sciences, Botanicheskaya 35, 127276 Moscow, Russia; losn1@yandex.ru (S.N.L.); savelievaek@ya.ru (E.M.S.); hotdogue@yandex.ru (D.V.A.); 2 General and Applied Botany, Institute of Biology, Universität Leipzig, Johannisallee 21–23, 04103 Leipzig, Germany; wolfram.brenner@uni-leipzig.de

**Keywords:** phosphotransmitter, multistep phosphorelay, histidine kinase, cytokinin signaling, transmembrane domain, phylogenetic analysis, molecular modeling

## Abstract

A new class of plant phosphotransfer proteins belonging to the multistep phosphorelay (MSP) system implicated in phytohormone cytokinin signaling was discovered based on large-scale bioinformatics methods. Unlike the canonical soluble nucleo-cytosolic forms, these proteins were predicted to have transmembrane (TM) domains and, apparently, should be localized on some kind of cell membrane. To date, 94 predicted TM-containing phosphotransmitter (TM-HPt) homologs were found in 62 plant species belonging to different clades, taxa, and groups of embryophytes: bryophytes, gymnosperms, and mono- and dicotyledons. The conserved HPt motif with phosphorylatable histidine was preserved in most of the TM-HPts under study, which allowed us to consider these proteins potentially active in MSP signaling. For the identified TM-HPts, a Bayesian analysis at the DNA level was performed, and a relevant phylogenetic tree was constructed. According to evolutionary relationships, plant TM-HPts were divided into two main groups corresponding to Arabidopsis AHP1-3,5,6, and AHP4 orthologs. Transcriptomic analysis confirmed the expression of most of the investigated TM-HPt-encoding genes. Their moderate-to-low overall transcription rate may be a consequence of inducible and/or tissue-specific expression. Using molecular modeling methods, a variety of potential spatial organizations of several such proteins are demonstrated. The ability of the uncovered TM domains to tether HPts to membranes was supported by molecular dynamic simulation. Possible roles of TM-HPts as modulators of the MSP signaling pathway and corresponding putative mechanisms of their action are suggested.

## 1. Introduction

Cytokinins are essential plant hormones that use a multistep phosphorelay (MSP) system for intracellular signal transduction [1,2,3,4]. These hormones are involved in diverse processes of plant growth and development, such as the formation of shoot meristem and leaves, cell division, vascular development, chloroplast differentiation, and leaf senescence, as well as responses to biotic and abiotic stresses. The cytokinin signaling is triggered by the specific binding of the cytokinin ligand to the receptor and leads to changes in gene expression [5,6,7,8].

The cytokinin signal transduction pathway involves a His-Asp phosphorelay similar to that found in bacterial two-component signaling systems [7,9]. However, unlike canonical bacterial signaling, which occurs via phosphotransfer between two protein residues, a conserved His residue in the sensor kinase and a conserved Asp residue in the receiver domain of the cognate transcription factor [10,11], in plants this phosphorelay is a multistep process, which includes three types of proteins: hybrid receptors with both histidine kinase (HK) and receiver domains, conserved histidine-containing phosphotransfer proteins or phosphotransmitters (HPts), and response regulators (RRs) containing a conserved phosphoaccepting Asp residue. In the commonly recognized scheme of cytokinin signal transduction, HPts play a special role linking the perception of cytokinins by membrane receptors to the activation of cytokinin-responsive transcription factors, type B RRs (RRBs), in the nucleus. The conserved His residue of the HPts accepts the «hot» phosphate from a conserved Asp residue of the activated cytokinin receptors, carries it into the nucleus, and phosphorylates there the conserved Asp residue of the RRB protein. Thereafter, phosphorylated RRBs become able to activate their target genes [3,12,13,14,15]. Therefore, HPts have a distinct function in the response to cytokinins in plant cells [16,17]. Suppression of the HPt genes reduced the cytokinin-triggered upregulation of sensitive genes, including type A RRs (RRAs), which are negative regulators of cytokinin signaling [18]. Insertional mutations in *AHPs* reduced the sensitivity of plants to the hormone in various cytokinin bioassays [19].

HPts are represented in plants by a family of small proteins with predicted histidine phosphotransfer activity [20]. Arabidopsis has a corresponding family of six members: AHP1–AHP5 that function as bona fide HPts, whereas APHP1/AHP6 is a pseudo-HPt (PHP) lacking the conserved phosphoaccepting His residue [21]. AHP1–AHP5 exhibit overlapping functions, mostly acting as positive cytokinin signaling compounds [19,22,23,24]. The complete blocking of phosphotransmitter gene expression in a quintuple *ahp1-5* mutant resulted in nonviable Arabidopsis seedlings [25]. By contrast, APHP1/AHP6 acts as a repressor of the cytokinin response pathway [26].

It should be noted that HPts and PHPs are apparently able to interact not only with proteins (sensor HKs, RRs) involved in cytokinin signaling, but also with other hybrid histidine kinases, including, for example, the Arabidopsis CKI1 kinase [27] and the ethylene receptor ETR1 [28,29]. Hence, HPts are versatile members of MSP, mediating signal output by various hybrid histidine kinases and input to a variety of RRB transcription factors [30]. In addition, the functions of PHPs are not the same across plant species. In rice, a member of the monocot family where PHPs are evidently also negative regulators of cytokinin signaling, disruption of *PHP* genes results in a subset of phenotypes distinct from those of the analogous mutants in Arabidopsis. This suggests that monocot and dicot PHPs can play non-identical roles in regulating plant growth and development [31]. Thus, some functions of both HPts and PHPs have yet to be uncovered.

In contrast to the remaining uncertainty in the peculiar functions of HPts and PHPs, the question about their localization seemed to have been resolved long ago. Initially HPts (in particular AHPs) were reported to be localized to the cytosol in unstimulated cells and accumulate in the nucleus upon cell exposure to exogenous cytokinin [16]. However, subsequent quantitative analysis revealed that the subcellular localization of the AHPs is constantly nucleo-cytosolic irrespective of the state of cytokinin signaling. Up to date it is believed that AHPs are soluble proteins constantly cycling between the nucleus and the cytosol [32]. This localization seems logical given the function of HPts in the MSP and, particularly, cytokinin signal transduction. Intriguingly, our recent bioinformatics analysis predicts the presence of transmembrane (TM) domains in some HPts. In this work, we carried out a global search and analysis of TM-containing plant HPts using bioinformatics methods. As a result, 94 putative plant TM-HPts were found, their domain architecture and biosynthesis at the transcription level were described, and a phylogenetic tree was constructed. Molecular models of a number of these proteins were built, and the ability of their TM domains to attach HPts to membranes was corroborated with molecular dynamic simulation. Based on the data obtained, the putative mechanisms of action of TM-HPts were suggested.

## 2. Results and Discussion

### 2.1. Search and Identification of Potential TM-Tethered Phosphotransfer Proteins

Previously, while studying and characterizing components of the cytokinin signaling pathway [15,33], we unexpectedly found that protein structure prediction servers identified TM domains in some HPts. Such a TM structure is not only uncharacteristic of plant HPts but also seems to contradict their known function. However, these data raised the issue of the existence of such proteins and their presence in various species. To address this issue, we have undertaken a large-scale search for potential TM-HPts using primarily the available sequenced genomes of land plants.

The first stage of the search for TM-HPts was carried out in a semi-automatic mode in the NCBI database (https://www.ncbi.nlm.nih.gov/, accessed on 19 March 2025). A description of the created program and its search algorithm is available in Section 3.1. This program allowed us to cover all plant species whose genomes were available in this database. TMHMM 2.0 (https://services.healthtech.dtu.dk/services/TMHMM-2.0, accessed on 21 March 2025) and Phobius (https://phobius.sbc.su.se/index.html, accessed on 25 March 2025) algorithms were used for the search of predicted TM domains.

The second stage of the search was carried out manually in the databases Phytozome (https://phytozome-next.jgi.doe.gov, accessed on 5 May 2025), PlantGenIE (https://plantgenie.org, accessed on 6 May 2025), Hornworts (https://www.hornworts.uzh.ch/en.html, accessed on 7 May 2025), and Fernbase (https://fernbase.org, accessed on 8 May 2025). As a result of the search, 94 potential plant HPts with predicted TM domains were found: 83, 8, 2, and 1 in the NCBI, Phytozome, PlantGenIE, and Hornworts databases, respectively.

Thus, the working set was formed from almost a hundred proteins. The number of TM domains and their positions were determined in each protein using CCTOP (https://cctop.ttk.hu, accessed on 16 May 2025) and Phobius servers. For each selected protein, the potential functionality in MSP was assessed. For this purpose, both the presence of an HPt active site (phosphorylatable histidine) and the putative binding surface (phosphorylation motif) were determined (Figure S1). The structure of the conserved phosphorylation motif, defined by aligning sequences of various known active HPts, is shown in Table 1. We have specified and extended this motif to 15 amino acid (aa) residues. The position of the conserved histidine (active site) was set to zero. The positions upstream and downstream of the conserved histidine are indicated by negative and positive numbers, respectively. HPts are considered active if they have phosphorylation motifs that strictly correspond to a conserved aa sequence. When only one aa residue deviated from the perfect motif, we still considered the corresponding proteins to be potentially active. In other cases, the conserved motif was assumed to be not preserved and the respective protein not a functional HPt (Table 2 and Table S1).

**Figure 1:**
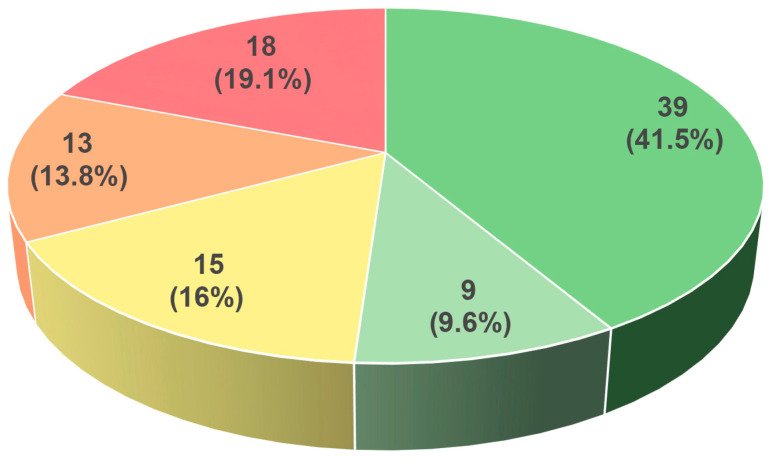
A diagram based on data in Table S1 demonstrates the distribution of TM-HPt proteins by color, that is, according to the degree of preservation of the phosphorylation motif. Green, light green, and yellow colors symbolize the presence of phosphorylatable histidine but varying degrees of conservation of the recognizable motif (see Table 1): complete, quasi-complete, and lower, respectively. Two remaining colors indicate the absence of phosphorylatable histidine, with high (orange) or low (red) conservation of the recognizable motif. Numbers of proteins/percentage of the total marked with respective colors are shown on the diagram.

The large diversity of proteins in the resulting set is noteworthy. They belong to 62 plant species, including representatives of Bryophyta (2 species), Gnetophyta (1 species), Coniferophyta (1 species), monocots (11 species), and dicots (47 species). Among 94 predicted TM-HPts, 39 are obviously active as classic HPts since they have both the conserved histidine and the phosphorylation motif within the HPt domain (Table S1). An additional 9 proteins can be classified as likely functional because they have a highly conserved phosphorylation site with only one aa substitution in the motif. Fifteen HPts harbor the conserved histidine, but their phosphorylation motif is altered at more than one residue. On the contrary 13 proteins preserved the HPt domain with an intact phosphorylation motif except for the conserved histidine replaced by another aa. In addition, 18 more proteins have lost both the active site and the corresponding motif (Figure 1). Collectively, more than half of the uncovered TM-HPts possess a perfect or quasi-perfect phosphorylation motif (shades of green), i.e., are potentially active.

In some plants, a single TM-HPt was found, and there were no similar proteins either within the same species or in related species of the same genus. Such a case may be illustrated by the *Juglans regia* genome, which encodes only one predicted TM-HPt (XP_018844524.2, Table 2). No other TM-HPt was found in the remaining *Juglans* genomes despite 36 species and variants of this genus being available in the NCBI genome database. At the same time, we found genera that include several species with putative TM-HPts. For example, in representatives of the *Salvia* genus, several TM-HPts were found per species, up to 9 in *Salvia splendens* (Table S1).

Most of the putative TM-HPts have non-transmembrane isoforms. This suggests that in the case of two or more transcript versions, mainly the canonical isoforms of the protein may be expressed. However, in some species, genes have been found that apparently encode only TM-HPt proteins (Table S2). In *Aquilegia coerulea*, *Brachypodium arbuscular*, *Brachypodium mexicanum*, *Carya illinoinensis*, *Cryptomeria japonica*, *Cucurbita pepo* subsp. pepo, *Gossypium barbadense*, *Gossypium darwinii*, *Manihot esculenta*, *Malus domestica* (XP_028945193.1), *Theobroma cacao*, *Vigna angularis*, and *Vigna radiata* var. radiata, predicted TM-HPts are encoded by genes that have only one transcript variant. The species *Salvia splendens*, in which two genes encode only TM-containing HPts, is of particular interest. In this case, one of the genes encodes a single TM-HPt variant, while another gene encodes three protein isoforms, each with predicted TM domains. Among proteins represented in Table S2, there are all variants of predicted TM-HPts with or without conserved histidine and/or phosphorylation motifs.

The polypeptide length of most plant TM-HPts is relatively short, which is typical for canonic cytosolic HPts. For example, the size of canonical *Arabidopsis* HPts lacking a TM domain is 154 ± 2 aa. The average size of proteins consisting of the group under study is 200 ± 7 aa, which is rather close to the size of cytosolic HPts. The shortest (100 aa) protein found belongs to *Linum usitatissimum*. Of particular interest are most large proteins from the studied cluster that often include additional domains. Along with the HPt domain, the *Malus domestica* protein RXH68939.1 is predicted to contain a Fatty Acid Desaturase domain larger than 200 aa. The *Camellia sinensis* protein THG10281.1 (Table S1) is defined as an auxin-responsive protein; its HPt domain is adjacent to an AUX/IAA domain, the size of the latter also exceeding 200 aa. KAK7845242.1 of *Quercus suber* is peculiar, as it is the only one in Table S1 that is predicted to contain two HPt domains. XP_024369173.1 of *Physcomitrium patens* can be considered the longest one among the compiled TM-HPts with a structural organization consisting only of a single HPt and TM domain. Our initial experiments with recombinant HPts harboring inserted TM domains from natural TM-HPts showed the ability of these domains to anchor the constructed proteins to cell membranes [34].

The number of TM domains within TM-HPts may vary from 1 to 5. CCTOP and Phobius servers predict different numbers and/or lengths of TM domains in some proteins, but the global statistics for both algorithms are similar. The median number of TM domains per protein according to both servers is 1. The median length of the TM domain is 19 aa, and the average one is 19.3 ± 0.3 and 19.5 ± 0.2 aa according to CCTOP and Phobius, respectively. This length is a bit shorter than the average length of TM domains of cytokinin receptors (21–22 aa) [35]. TM helices are located in the proteins under study either at the N- or at the C-terminus, in some cases even in the middle.

It should be noted that functional phosphotransfer proteins containing both TM and HPt domains are quite common in bacteria. We were able to find thousands of such proteins in the prokaryotic databases. As a rule, they differ from plant TM-HPts in their large sizes, which may exceed 1500 aa. Such a size is often due to a number of diverse functional domains along with HPt and TM ones [30]. For instance, the well-known TM histidine kinase RcsC of *E. coli* transfers its signal first to the membrane-bound phosphotransfer protein RcsD (890 aa), which in turn transmits it to the transcription factor RcsB [36]. Notably, among bacterial phosphotransfer proteins, there are also those very similar to plant TM-HPts both in size and structure, for example, proteins WP_083661941.1 of *Actinophytocola xanthii* (168 aa) and WP_244434644.1 of *Afipia* sp. P52-10 (184 aa) with predicted structures consisting of one HPt domain and one TM domain only.

### 2.2. Phylogenetic Analysis of TM Phosphotransfer Proteins

**Figure 2:**
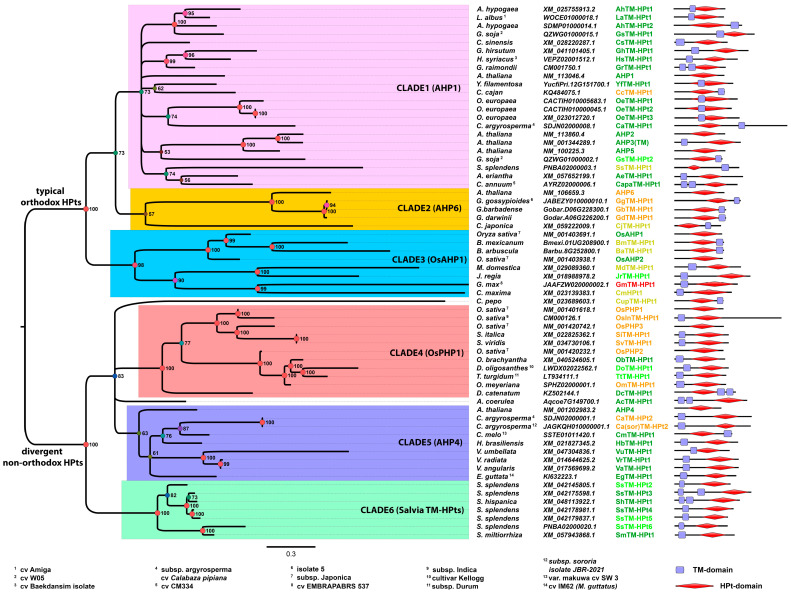
Phylogenetic tree of plant TM-HPts. This tree was constructed using MrBayes-3.2.7. The columns on the right provide the following reference information (from left to right): plant species names, transcript accession IDs, trivial names, and schemes of domain composition in TM-HPts. In the third column, the color code is the same as in Table 2 and Table S2.

To clarify evolutionary relationships between potential TM-HPt proteins, we performed a phylogenetic analysis. It should be noted that the phylogenetic analysis of such short sequences is hardly possible due to their rather high similarity. The use of protein sequences and algorithms of software like MEGA (https://www.megasoftware.net/, accessed on 20 May 2025) resulted in low confidence phylogenetic trees. We carried out the analysis with MrBayes (https://nbisweden.github.io/MrBayes, accessed on 23 May 2025) using not aa but coding nucleotide sequences. This approach gave highly reliable results. Only sequences of angiosperms were used in the analysis, since adding representatives from other groups significantly complicates (and currently does not allow) obtaining a tree of sufficient quality. The list of analyzed proteins also excludes the apparently inactive TM-HPts marked red in Table S1. The resulting phylogenetic tree is presented in Figure 2.

The overall tree topology was similar to that generated previously [37]. As in the mentioned work, we constructed a tree only for representatives of angiosperms due to the difficulties in establishing the exact position in the tree of the branches of non-angiosperm HPts. Each transcript/protein (Table S3) represented in the tree (Figure 2) corresponds to a distinct gene. Our tree has two large branches, which we designated as typical orthodox HPts and divergent non-orthodox HPts. According to the reference species *Arabidopsis thaliana* and *Oryza sativa* subspecies Japonica, the first group includes orthologs of AHP1-3,5,6, and OsAHP1 and 2, while the second group includes orthologs of AHP4 and OsPHP1-3. It should be noted that the Arabidopsis HPt AHP4 stands apart, as it has small structural and significant functional differences from other HPts of this plant; in particular, it hardly supports canonical phosphotransfer into the nucleus [38]. Each branch in turn is divided into three clades. We named each clade after a typical representative or, in the case of clade 6, according to a particular plant genus (*Salvia*). Clades 1 and 2 do not contain representatives of the grasses from monocots. Clade 2, however, consists only of PHPs—orthologs of phosphorelay inhibitor AHP6 from Arabidopsis. Among members of Clade 3, there is only one potentially active HPt of *Juglans regia*. Most likely, dicots tend to reduce genes belonging to Clade 3, as indicated by the longest branch length (accelerated mutagenesis) in the entire tree for the remaining three dicots in this clade, *Malus domestica*, *Glycine max*, and *Cucurbita maxima*. Clades 4 and 5 include representatives of monocots and dicots, respectively. In the fourth clade, not all proteins are PHPs, as is typical for *Oryza sativa* subspecies Japonica. The *Oryza brachyantha* member is an apparently active HPt. Similarly, clade 5 contains both active and pseudo HPts.

Notably, HPts of the genus *Salvia* were allocated in a separate clade, which has not been recognized before. For example, in the work [37], where the phylogeny of cytokinin signaling components was considered in detail, this clade was absent, since the sample did not include the corresponding plant species. According to our preliminary data, this clade is present in potato, tomato, *Hevea brasiliensis,* and a number of other plants but is still absent in most species. In general, it can be assumed that the TM domain is found in all known phylogenetic clades in angiosperms, most often single and upstream of the HPt domain. At the same time, no reliable relationship was found between the phylogenetic status of the TM-HPt and the number and location of TM domains in the protein. Apparent distinctive traits, such as those in clades 2 and 6, are preferably explained by the close relationship of the plant species involved. This means that in this case the most likely scenario is that this gene series originated from a single TM-HPt gene in an evolutionarily close ancestor of the group at the origin of the genus or at the formation of subgenera within the genus.

The emergence of TM-HPt lines at the stage of genera or subgenera formation is observed in several cases when species of one genus form clusters in which species from other genera are absent. This is observed in the genera *Salvia* (Clade 6), *Vigna* (Clade 5), *Brachypodium* (Clade 3), *Setaria* (Clade 4), and *Gossypium* (Clade 2). It is interesting that in the genus *Gossypium,* TM-HPts appeared not only in clade 2 (GgTM-HPt1, GbTM-HPt1, and GdTM-HPt1), but also in clade 1 (GhTM-HPt1 and GrTM-HPt1). However, a representative from *Hibiscus sinensis* “wedges” itself into the latter clade. In several species, TM-HPts seem to independently arise several times. In *Arachis hypogea*—in Clade 1 (AhTM-HPt1 and 2), in *Salvia splendens*—in Clade 1 (SsTM-HPt1) and many in its own Clade 6 (SsTM-HPt2-6), and in *Glycine soja*—in Clade 1 (GsTM-HPt1 and 2). Thus, we can conclude that the evolution of phosphotransfer proteins towards the formation of TM-HPts in angiosperms, and, taking into account the non-angiosperm species not included in the tree, in all land plants as a whole, is active and occurred in a largely independent way in different genera.

### 2.3. Transcriptomic Studies of TM-HPt Genes

**Figure 3:**
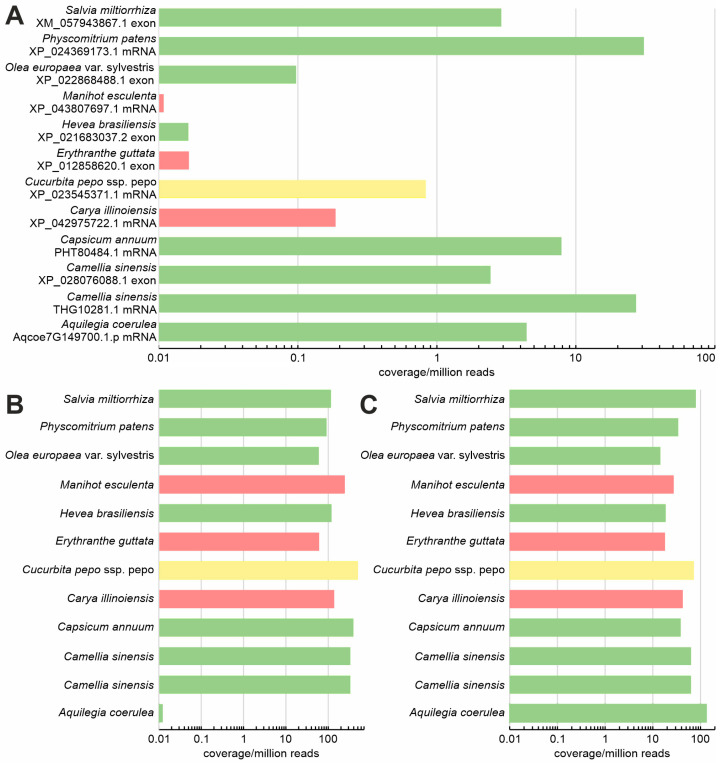
Expression of the transcripts encoding TM-HPt proteins (**A**) and of the orthologs of the two *Arabidopsis thaliana* reference genes *UBC10* (AT5G53300, (**B**)) and *PP2AA2* (AT3G25800, (**C**)). Expression of the transcripts is estimated by the maximum coverage, plotted as coverage per million reads. For each TM-HPt transcript, the respective protein ID is given. In the case of a single transcript, the entire mRNA was used for maximum coverage calculation (identified as “exon”). The color code of the bars in (**A**) is the same as in Table 2, Tables S1 and S2. For reader convenience, the color of the bars of the reference genes in (**B**,**C**) is the same as in (**A**).

The question of whether transcripts encoding TM-HPt proteins are expressed was addressed using a transcriptomic approach in 12 plant species covering various classes of TM-HPt proteins grouped in Table S4. The analysis revealed that most of the investigated transcripts encoding functional TM-HPt proteins are, in most cases, rarer in comparison to two frequently used reference genes (Figure 3). The highest expression was found in the transcript of the *Physcomitrium* TM-HPt, which reached a moderate transcription level comparable to the PP2AA2 reference transcript, closely followed by the transcript encoding the *Camellia sinensis* protein THG1028.1. Of eight functional TM-HPt transcripts investigated (green color), the one of *Hevea brasiliensis* (RNA extracted from latex) can be regarded as absent. Two of the three annotated transcripts encoding non-functional TM-HPt proteins (red color) are virtually not transcribed as well. The transcript of *Cucurbita pepo*, whose encoded protein contains an active phosphorylation site but a significantly altered motif (yellow bar), was at best marginally transcribed.

In summary, it can be stated that transcripts encoding functional TM-HPt proteins are expressed, but most of them at rather low levels (Figure 3). The highest transcription rate (maximum coverage between 1 and 100 per million reads) was found in transcripts encoding proteins with a fully functional phosphorylation motif (green bars). Intriguingly, two of the three annotated transcripts encoding non-functional TM-HPts (red bars) are virtually not expressed. These non-functional genes may have become pseudogenes. The only “red” gene (from *Carya illinoiensis*) that appears to be marginally expressed encodes a protein completely lacking the conserved phosphorylation motif. This severely mutated protein may no longer take part in MSP signaling. The rather high expression level of the *Physcomitrium* TM-HPt transcript may indicate a more prominent role of TM-HPts in early land plants; however, more TM-HPt transcripts of mosses and pteridophytes would have to be studied to corroborate this notion.

In fact, the moderate-to-low transcript content of many TM-HPt genes in the whole plant organism may be indicative of the organ- and/or tissue specificity of these genes. It also cannot be ruled out that the expression of this group of genes may be confined to a specific stage of plant development or controlled by inducible promoters. All of this was not investigated here because the selection of RNA sequencing datasets for non-model species is limited. The purpose of this approach was rather to show whether TM-HPt transcripts do exist at all and how widespread they are among land plants. Further detailed studies of the genomes and transcriptomes of the above-mentioned plant species will clarify this issue.

### 2.4. Molecular Modeling of TM-HPts

To evaluate the possible functionality in more detail as well as to investigate the spatial organization of the studied proteins, structural models of different TM-HPts were built. Their orientation in the membrane was predicted using the PPM 3.0 service (https://opm.phar.umich.edu/ppm_server3_cgopm, accessed on 3 June 2025). Some of these models were optimized for positioning in the membrane profile since they were initially constructed without appropriate constraints.

We used the canonical soluble HPt 3D shape (AHP 1–3, 5) as a reference when comparing the resulting TM-HPts models. The canonical structure of HPts involved in MSP signaling consists of six α-helices. Three out of the six α-helices (α2, α3, and α4) are involved in the formation of the interaction interface with the receiver domain (RD) of the receptor kinase [39]. The protein–protein interaction interfaces of the HPt homodimer and the HPt–HK_RD_ heterodimer, overlap significantly [15].

**Figure 4:**
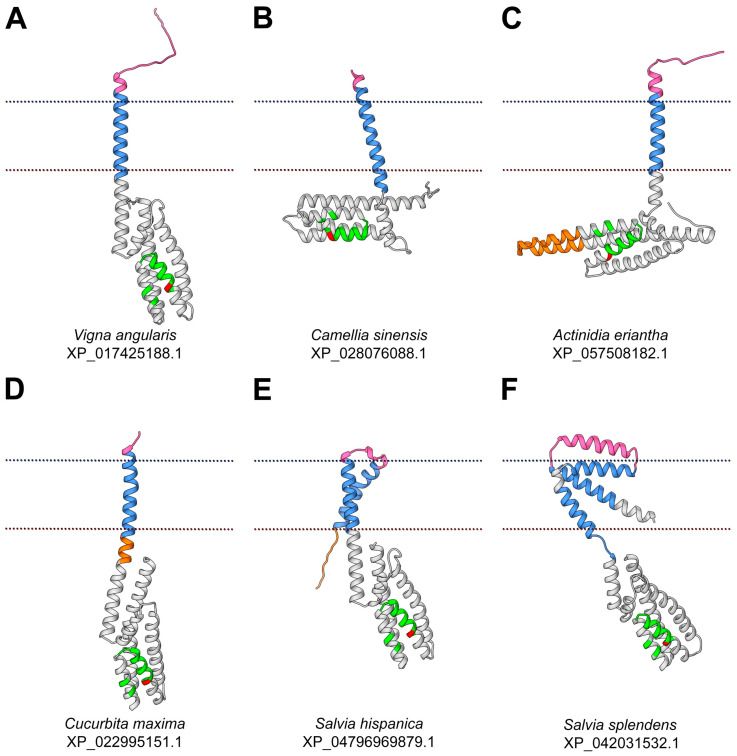
Selected molecular models of TM-HPts categorized by topology and folding features. Each model represents one of the structural groups into which it has been categorized. (**A**) *Vigna angularis* XP_017425188.1 belongs to group 1; (**B**) *Camellia sinensis* XP_028076088.1—group 2; (**C**) *Actinidia eriantha* XP_057508182.1—group 3; (**D**) *Cucurbita maxima* XP_022995151.1—group 4; (**E**) *Salvia hispanica* XP_047969879.1—group 5; (**F**) *Salvia splendens* XP_042031532.1—group 6; The TM segment is highlighted in blue, the cytosolic insertion in orange, the extracytosolic region in pink, the conserved motif is shown in green, and the phosphoaccepting histidine in red.

The diversity of topology and folding of the obtained models of different TM-HPts was in the number of TM domains, the completeness of the canonical HPt part, the spatial arrangement features, and the size of the extracytosolic part. We use the term «extracytosolic» for protein regions located on the side of the membrane opposite to the canonical HPt moiety. In this case, it does not matter on which side of the membrane the prediction software placed this fragment. This is due to the fact that classical HPts have been shown to preferentially localize in the cytosol and the nucleus, but not in the apoplast [40,41]. We divided all the resulting models into several groups (Figure 4).

In proteins classified in the first group, the basic HPt part is mostly preserved, the TM domain is a direct elongation of the N-terminal α1-helix of the HPt moiety, and the extracytosolic fragment is mostly unstructured (Figure 4A). Such proteins are *Vigna radiata* XP_014500111.1 and *Vigna angularis* XP_017425188.1. In both *Vigna* spp. proteins, the canonical HPt part is preserved completely, and the extracytosolic part is short, mostly unfolded, and includes an N-glyco motif according to Protter (http://wlab.ethz.ch/protter/start/, accessed on 6 June 2025). The N-glyco motif is the site of N-glycosylation, the biochemical process of attaching a glycan to the *N*4 atom of an asparagine residue. N-glycosylation has a structural function, affects protein stability and solubility, protects proteins from aggregation, and can mediate signal transduction in cells [42,43].

The second group of the HPt set includes proteins whose α1-helix of the canonical HPt part is significantly shortened and continuously transitioned to the TM or completely replaced by the TM helix (Figure 4B). *Camellia sinensis* XP_028076088.1, *Populus trichocarpa* XP_024465836.1, *Malus domestica* XP_028945193.1, and *Actinidia eriantha* XP_057508184.1 belong to this type. The extracytosolic part of this protein group is variable. In *Camellia sinensis* XP_028076088.1, it is a short continuation of the TM helix (5 aa in total). In *Populus trichocarpa* XP_024465836.1, it is a long α-helix (26 aa). In *Malus domestica* XP_028945193.1 there is a large, unfolded region 42 residues long containing an N-glyco motif.

*Actinidia eriantha* XP_057508182.1 (Figure 4C) belongs to the third structural group. Together with *Olea europaea* XP_022868488.1, these two proteins have an insertion within the canonical HPt part itself. *Actinidia eriantha* XP_057508182.1 has an insertion caused by the elongation of the α5 and α6 helices of the HPt fragment. In *Olea europaea* XP_022868488_1, atypically arranged TM regions localize not at the termini of the canonical HPt domain but in the middle of it as an insertion between α3 and α4 helices.

In the fourth structural group (Figure 4D), the cytosolic portion of the α1-helix is elongated rather than shortened, i.e., there is an additional fragment between the canonical HPt part and the TM region. The model of *Cucurbita maxima* XP_02299995151.1 shows such an elongation. In *Cucurbita maxima* XP_02299995151.1, the N-terminal region, the TM domain, and the elongated α1 of the canonical part represent a single continuous α-helix. Again, the HPt domain here contains an N-glyco motif.

The TM-HPts of *Salvia* spp. are of particular interest in terms of structural diversity. Their HPt proteins include one to three TM domains. The considered *Salvia hispanica* proteins have two N-terminal TM domains connected by a short linker (Figure 4E) and belong to the fifth structural group. The difference between XP_047969881.1 and XP_047969879.1 is in the 17 aa shortening of the C-terminal α6-helix of the canonical HPt part of the first protein.

Nine TM-HPts were found in *Salvia splendens*; three of them have three TM domains, and six others have one TM domain, respectively. The spatial shape of all three proteins with three TM helices (XP_042031532.1, XP_042031534.1, and XP_042031535.1) is similar, which allowed us to put them into a sixth group (Figure 4F). Between the first and the second TM motifs there is a helix-shaped insertion comparable in size to the TM segments. The third TM domain is connected to the canonical HPt part flexibly via a loop. These proteins are distinguished by the length of the C-terminal α6-helix of the canonical part. In XP_042031532.1, this part is full-sized, whereas in XP_042031534.1, the C-terminal α-helix is reduced by about a half, and in XP_042031535.1, the canonical part comprises only five α-helices.

Among the obtained models, which are not considered in detail in this paper, a few more groups can be distinguished. First, these are TM-HPts with a localization signal sequence in the extracytosolic part. These include XP_025611698.1 of *Arachis hypogaea* and XP_047160793.1 of *Vigna umbellata*. Second, these are phosphotransfer proteins with a single TM domain but located at the C-terminus rather than at the N-terminus. Such HPts may be exemplified by those of *Picea abies* MA_8815334g0010 and *Gossypium darwinii* Godar.A06G226200.1.p. Finally, there are proteins that have two predicted potentially functional domains. *Quercus suber* KAK7845242.1, according to the model, includes two predicted HPt domains separated by three TM domains, a small extracytosolic region, and several cytosolic domain extensions. *Camellia sinensis* THG10281.1 has a canonical HPt domain with all six preserved α-helices located at the N-terminus, as well as a PB1 domain typical for auxin signaling proteins at the C-terminus.

### 2.5. Molecular Dynamics Simulation of TM-HPts in Artificial Membrane

To assess whether the newly identified TM-HPt proteins are indeed capable of being integrated into biomembranes, several protein models were embedded into an artificial membrane model mimicking the ER membrane composition (according to [44]) using the Yasara Structure software (version 22.9.24) [45]. The following TM-HPts were selected for this study: *Actinidia eriantha* XP_057508182.1, *Cucurbita maxima* XP_022995151.1, *Camellia sinensis* XP_028076088.1, *Salvia hispanica* XP_047969879.1, *Salvia splendens* XP_042031532.1, *Vigna angularis* XP_017425188.1, and *Arabidopsis thaliana* NP_001330681.1 (AHP3 isoform with predicted TM domain). All but one of the selected proteins possess a perfect phosphorylation motif (marked green in Table 2 and Table S2), with the exception of the protein of *Cucurbita maxima* XP_022995151.1 (marked yellow). The *Arabidopsis thaliana* cytokinin hydroxylase CYP735A2, for which transmembrane localization in the ER has been predicted [8,46], was selected as a positive control. CYP735A are members of the family of eukaryotic cytochrome P450 proteins, which were experimentally shown to localize in the ER membrane [47,48].

For each protein model, including the control, a 50 ns molecular dynamics (MD) simulation was performed using Yasara Structure software. After that, the binding energy of proteins interacting with the membrane was calculated using Yasara with the PBS method. According to the Yasara Structure protocol, more positive values mean stronger binding.

**Figure 5:**
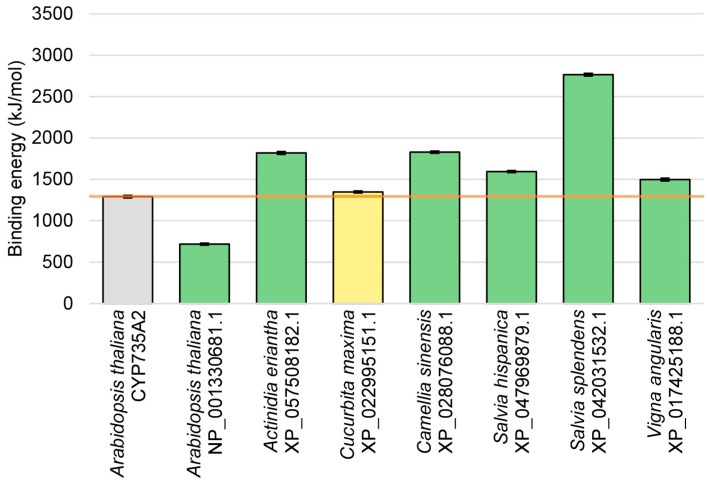
The calculated average energy of putative transmembrane HPts binding to ER-mimicking membrane during 50 ns MD simulation. According to the Yasara Structure protocol, more positive values mean stronger binding. Data are presented as means with standard errors (SE). The color code of the bars is the same as in Table 2, Tables S2 and S3. Grey bar and orange line show the level of the positive control.

The analysis showed that the energy of interaction with the membrane of almost all proteins studied was higher than or comparable to that of the membrane-bound CYP735A2 (Figure 5). This can be seen as strong evidence for transmembrane localization of these HPts, presumably in the ER membrane. Interestingly, TM-HPt of *S. hispanica* with two TM domains showed a membrane-binding energy comparable to that of HPts with a single TM domain, whereas *S. splendens* protein with three TM domains exhibited a significantly higher membrane-binding energy.

At the same time, the *Arabidopsis thaliana* AHP3 isoform version (NP_001330681.1) with the predicted TM domain shows much less binding energy than the CYP735A2 control and other analyzed HPts. Although some affinity of NP_001330681.1 toward the membrane is indeed predicted by the corresponding software, the almost twofold difference between this protein (717.461 kJ/mol) and the control CY735A2 (1291.425 kJ/mol) indicates a clear inferior interaction of NP_001330681.1 with the membrane. This result is in agreement with our recent experimental data which confirmed the membrane localization of tea *C. sinensis* TM-HPt but did not detect the binding of the Arabidopsis version of TM-HPt to cell membranes.

### 2.6. Possible Functional Meaning for the Presence of the TM Domain in Plant HPts

**Figure 6:**
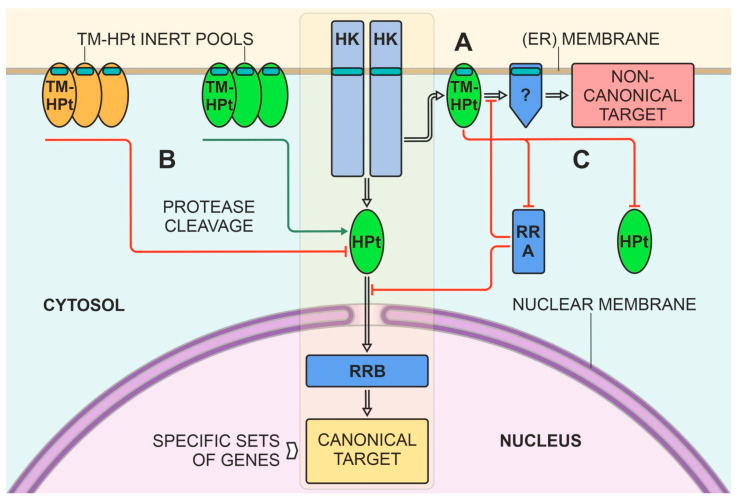
Hypothetical effects of TM-HPts on plant histidine kinase signaling. Canonical signal transduction is marked by a yellow background. HK, histidine kinase; HPt, phosphotransmitter; RRB, nuclear response regulator type B; RRA, response regulator type A. Light stripes in the figures indicate TM domains. Transparent arrows mean steps of signal transduction; colored arrows mean the object’s movements and interactions; green arrows show up-regulation, while red arrows show down-regulation of MSP. (**A**) a case of alternative non-canonical target, probably membrane-bound too. (**B**) a case of formation of inert pools from TM-HPts on (ER) membrane, functional TM-HPts marked green, inhibitory TM-HPts marked orange. These pools can become soluble and participate in signaling upon endoprotease cleavage. (**C**) a case of heterodimer formation of TM-HPt with soluble HPt or RRA.

The functional meaning of the TM domains in plant HPts obviously depends primarily on the structural features of the TM-HPt itself. As this work has shown, the ability of TM-HPts to specifically transfer a “hot” phosphate varies over a wide range, where on the one hand there are proteins with a preserved canonical HPt domain and an impeccable phosphorylation site, while on the other hand there are proteins with heavy mutations of both. It is natural to assume that the functional role of proteins with opposite structural features will most likely also be different. Membrane proteins with a conserved histidine capable of transferring the signal in the form of mobile phosphate can create alternative signaling branches distinct from the canonical MSP signaling to the nucleus and directing it to certain membrane-bound targets (Figure 6A). This hypothesis is supported by our preliminary data on the presence of not only transmembrane HPts but also transmembrane RRs in plants. Moreover, the suggested structural diversity of TM-HPts argues for putative multivariate targets in the cell beyond the genetic material. As an analogy, we can refer to bacterial HPts, many of which are membrane-associated proteins with complex multidomain structures [30].

Another possibility for the noticeable influence of TM-HPts on the action of the MSP system is that these proteins may form a kind of inert pool on membranes (Figure 6B). Such a pool can be, in principle, capable of rapidly switching to an active state in the case of cleavage of the polypeptide chain at some point between the TM and HPt domains. Such cleavage rendering these phosphotransfer proteins soluble is possible upon induction of the expression and/or activity of one or more specialized endoprotease(s). Considering the huge number of protease genes identified in plants (>650 and >800 in rice and Arabidopsis, respectively, [49]), such a scenario seems quite plausible. Indeed, there are several examples of re-localization of membrane-tethered proteins from the ER to the nucleus. These proteins represent various plant transcription factors with typical TM domains, which are exposed to endoprotease cleavage under stress conditions ([50] and refs therein). Notably, whether the effect on signaling would be positive or negative may directly depend on the main characteristics of the cleaved TM-HPt. In the case of cleavage of active proteins (marked green), the MSP pathway should be enhanced. Conversely, in the case of cleavage of TM-HPts lacking the conserved histidine (marked orange), it should be weakened, as these proteins may be inhibitors of MSP signaling.

Another option for the negative effect of TM-HPt on phosphorelay signaling may be the preserved ability of membrane-bound HPt to dimerize (Figure 6C). It has been shown experimentally that canonical HPts can form heterodimers with paralogs [51]. Dimerization of the TM-HPts and the canonical ones can sequester the mobile cytosolic HPts, reducing their active concentration in the cell and thereby weakening the canonical MSP. The interaction of TM-HPt and RRAs may also play a role in the regulation of the CK signal (Figure 6C). The existence of functional cytosol-localized RRAs has been demonstrated experimentally [16,32,52,53].

Thus, TM-HPts can act as modulators of the strength and specificity of plant sensory histidine kinase signaling, including directing MSP signaling to new and yet unknown, non-canonical targets. The presumably independent origin and structural diversity of TM-HPts suggest their different functions in land plants. In general, this work has opened up new, previously unknown areas of science that can lead to a breakthrough in our knowledge of the molecular mechanisms of action of phytohormones (cytokinins) and MSP-based plant signaling systems.

## 3. Methods

### 3.1. Bioinformatics Search for Phosphotransmitters with TM Domains

The algorithm of a program specially created for TM-HPt search is shown schematically in Figure S2. Based on the NCBI Protein Reference Sequences database, the program performed an automatic search and alignment using NCBI BLAST (https://blast.ncbi.nlm.nih.gov/Blast.cgi, accessed on 9 June 2025) [54] (protein-protein BLAST algorithm, refseq_ and nr_ databases) of all TM-HPt homologs available in the NCBI database. The retrieved sequences were automatically passed through the «one species—one protein» filter (SINGLESPEC_FILTER, see Figure S2). This filter uses the species name of the protein contained in the FASTA sequence metadata. All non-plant proteins were manually removed from the resulting pool of sequences (STAGE-1 OUTPUT). Selected plant sequences were used to repeat the first step (search and alignment of homologs). This time each of the plant-belong sequences from STAGE-1 OUTPUT in turn was used as a query sequence. The number of output sequences was limited to 100,000 proteins. Repeated proteins were automatically removed. The remaining data were automatically screened using the TMHMM 2.0 [55] and Phobius [56] algorithms for the presence of predicted TM domains.

All proteins for which at least one of the services predicted the presence of TM domains were manually checked using the CCTOP service [57] to exclude proteins from the working sample whose detection of a TM domain is an artifact of a specific predictor program. Upon this check, another small part of the proteins was eliminated. Searches in the databases Phytozome, PlantGenIE, Hornworts, and Fernbase were performed manually using the BLAST(p) tool (https://blast.ncbi.nlm.nih.gov/Blast.cgi, accessed on 9 June 2025). All found HPts passed through the TM filter described above. All selected plant proteins with predicted TM domains were checked on the InterPro website (https://www.ebi.ac.uk/interpro, accessed on 12 June 2025) [58] for the presence of HPt domain(s). If the HPt domain of a protein was defined by at least one of the databases integrated into InterPro, we kept it in our set.

### 3.2. Phylogenetic Analysis

We have analyzed the nucleotide protein-coding sequences of 64 genes. Each such sequence corresponded to one gene. They were aligned using Clustal Omega (https://www.ebi.ac.uk/, accessed on 12 June 2025). Phylogenetic trees were constructed using MrBayes-3.2.7. Clustal alignments were used as input, and Bayesian MCMC phylogenetic trees were constructed based on Markov chain Monte Carlo simulation with the general time reversible (GTR) nucleotide substitution model and site-rate variation drawn from a discrete gamma distribution with six classes. 1,000,000 generations were taken for reaching a standard deviation of split frequencies below 0.01. The resulting tree was visualized by FigTree v1.4.4 (http://tree.bio.ed.ac.uk/software/figtree, accessed on 14 June 2025).

### 3.3. Determination of the Expression of TM-HPt-Encoding Transcripts

To determine whether annotated transcripts encoding TM-HPt proteins were expressed, we employed a large-scale transcriptomic approach. The RNA-sequencing datasets used are listed in Table S4. RNA-sequencing data of large experiments were downloaded from the NCBI SRA database using the prefetch tool of the NCBI SRA Tools suite [59]. The SRA files were extracted to FASTQ files using the fasterq-dump tool. The FASTQ files were quality-checked using the FastQC (https://www.bioinformatics.babraham.ac.uk/projects/fastqc/, accessed on 18 December 2025) implementation in Unipro UGENE [60,61,62].

The reads were mapped to the templates using Rsubread [63]. Templates for mapping the reads were generated as FASTA files containing the annotated mRNA encoding the TM-HPt, or—if not available—the corresponding genomic sequence. In addition, the mRNAs of the orthologs of the *Arabidopsis thaliana* genes At5G53300 (encoding UBC10) and AT3G25800 (encoding PP2AA2) were included as references.

The resulting BAM files were merged using UGENE. The merged BAM files were imported into UGENE to visualize the mapped reads. UGENE was used to determine the number of reads mapped. The maximum number of reads was used as an estimate for expression of the TM-HPt transcripts. If the TM-HPt transcript was a splicing variant, only the TM-HPt-specific part of the transcript was considered. This is marked with “exon” in the tables and figures. To normalize for differing sequencing depths between the species, the number of mapped reads per million total reads was calculated and plotted in the resulting graph. The entire process from downloading the SRA files to mapping with Rsubread was automated in a custom-made R script using the metadata of the SRA dataset as a starting point.

### 3.4. Molecular Modeling

Molecular modeling by the de novo method was performed using the IntFOLD (version 7.0) web service (https://www.reading.ac.uk/bioinf/IntFOLD/, accessed on 24 June 2025) [64]. The best models were selected based on both the IntFOLD score and the relevance of protein folding and topology to potential localization in the membrane. The pLDDT values of selected initial models are presented in Figure S3. The models were optimized in Yasara Structure software (version 22.9.24) [45] using the md_refine macro. This macro runs MD simulations for a 500 ps model using the protocol described in [65]. The pH value was set to 7.4. Prediction of TM regions and signal peptides/sequences was based on Phobius [56] implemented in the Protter web service (http://wlab.ethz.ch/protter/start/, accessed on 6 June 2025) [66], which was also used for identification of N-glyco motifs. Orientation of modeled proteins in membrane was predicted in PPM 3.0 Web Server (https://opm.phar.umich.edu/ppm_server3_cgopm, accessed on 3 June 2025) [67] and Yasara Structure. Models were additionally optimized and visualized in UCSF Chimera software (version 1.14) [68].

### 3.5. Molecular Dynamics Simulation in Artificial Membrane

Embedding of protein models into the membrane and molecular dynamics simulation were performed using Yasara Structure (version 22.9.24) [45] with the “md_runmembrane” macro. For ER membrane mimicking, the following ratio of membrane components was used: 27% phosphatidylethanolamine (PEA or PE), 50% phosphatidylcholine (PCH or PC, also known as POPC), 3% phosphatidylserine (PSE or PS), 7% phosphatidylglycerol (PGL or PG), and 13% cholesterol (CLR) for both layers of the lipid bilayer, in accordance with [44], values rounded.

Extension of the cell on each side of the protein in the membrane plane was set to 15 Å. Extension of the cell along the third (water) axis was set to 10 Å. Protonation states were assigned according to pH 7.4, and the simulation cell was filled with water, 0.9% NaCl, and counter ions [65,69,70]. Simulation was run for 1 nanosecond of the equilibration stage and 50 nanoseconds of the production stage using the AMBER14 force field [71], with Lipid17/GAFF2/AM1BCC parameters for nonstandard residues. Simulation temperature was 298 K, and pressure was 1 bar. The main simulation (production stage) was run with an 8.0 Å cutoff for non-bonded real space forces. The equations of motion were integrated with a multiple time step of 2.5 fs (fast protocol). The save interval for snapshots was 100 ps.

Trajectories were analyzed using the “md_analyzebindenergy” macro in YASARA Structure. Binding energies were analyzed using the Poisson–Boltzmann (PBS) method. The method is equal to ‘MM/PBSA’, just without the entropy term from normal mode analysis. The temperature was 298 K, and the force field was AMBER14.

## 4. Conclusions

A global search for a special class of phosphotransfer proteins harboring TM aa segment(s) and belonging to the MSP system of plants has been performed using large-scale bioinformatics methods. The MSP system plays a crucial role in intracellular signal transduction of cytokinins, classical plant hormones. The analysis of more than 120 sequenced plant genomes revealed about a hundred predicted phosphotransfer proteins with the unconditional presence of TM domains, identified by rigorous independent algorithms (Table 2 and Table S1). Totally, about a hundred of such proteins from 62 species were uncovered, most of them with preserved functional domains and thus considered potentially active. Phylogenetic analysis has divided these TM-HPts into two main groups, one representing orthologs of Arabidopsis’ AHP4, while the other represents orthologs of all other rather uniform HPts (AHP1–3, 5) of this species. Although most of these proteins are isoforms of canonical soluble HPts encoded by distinct transcript versions, about two dozen genes in 13 plant species were found expressing a single mRNA version encoding TM protein (Table S2). Together with transcriptomic data that most transcripts encoding TM-HPt are likely produced in the cell in vivo (Figure 3), this removes virtually all doubt about the actual existence of such proteins in the cells of many plants. The structural models of the revealed proteins were built using molecular modeling methods. A wide variety of model structures and their high affinity toward ER-mimicking membrane models have been demonstrated using the molecular dynamics method (Figure 4 and Figure 5). This result was further confirmed experimentally in a study where particular TM-HPts were indeed found in the cellular membrane fraction [34]. Further experimental verification of the results of the current in silico analysis is possible using various methods like confocal microscopy of labeled proteins, testing phosphotransfer activity, generation of transgenic plants with mutated target genes, and others.

The discussion of all obtained data led to the understanding that TM-HPts can influence, either positively or negatively, the canonical MSP signaling. New, as yet unexplored signaling pathways also cannot be ruled out. This work laid the foundation for a targeted study of non-canonical membrane branches of the MSP pathway in many plant species. Among the latter, there are economically valuable species such as rice, wheat, soybean, sunflower, cotton, etc. This indicates the particular importance of this newly discovered area of research not only for fundamental science but also for its practical application.

## Figures and Tables

**Table 1:** Conserved His phosphorylation motif in active HPts. The sequence of typical aa adjacent to the phosphorylatable His residue is indicated, along with their helix and space localization.

Position aa Number		Typical aa on this Position	α-Helix	Facing Outside or Inside
H Set to 0	in AHP2			
−1	81 (V)	V, M	α4	in
0	82 (H)	H *	α4	out
1	83 (Q)	Q	α4	out
2	84 (L)	L, F, Y	α4	in
3	85 (K)	K	α4	out
4	86 (G)	G	α4	out
5	87 (S)	S	α4	out
6	88 (S)	S	α4	in
7	89 (S)	S, A, T	α4	out
8	90 (S)	S	α4	out
9	91 (V)	V, I	α4	out
10	92 (G)	G	α4	in
11	93 (A)	A	α4	in
19	101 (V)	V, I, A, L, T, S, M	α5	out
22	104 (K)	K, R, Q	α5	out

* Phosphorylatable histidine.

**Table 2:** List of some potential TM-HPts and their main characteristics.

Plant Species	Protein ID	Database	Protein Length (aa)	Predicted TM Position:		Active Site	Binding Motif
				CCTOP	Phobius		
*Actinidia eriantha*	XP_057508182.1	NCBI	211	17–34	20–39	+	+
*Anthoceros punctatus*	Apun_evm.model. utg000023l.837.1	Hornworts	241	198–215	199–219	+	+
*Aquilegia coerulea*	Aqcoe7G149700.1.p	Phytozome	224	25–52	12–32 38–57	+	+
*Arachis hypogaea*	XP_025611698.1	NCBI	159	14–33 52–67	51–68	+	+
*Camellia sinensis*	XP_028076088.1	NCBI	164	7–32	6–32	+	+
*Glycine soja*	RZB63908.1	NCBI	206	90–109	90–112	+	+
*Olea europaea* subsp. europaea	CAA3000307.1	NCBI	177	69–96	57–76 82–99	+	+
*Oryza brachyantha*	XP_040380539.1	NCBI	156	6–24	6–28	+	+
*Physcomitrium patens*	XP_024369173.1	NCBI	425	77–101 161–176	37–55 76–98 159–176	+	+
*Salvia splendens*	XP_042031532.1	NCBI	236	12–29 81–98	12–30 58–75 82–101	+	+
	XP_042031534.1	NCBI	219	12–29 81–98	12–30 58–75 82–101	+	+
*Vigna angularis*	XP_017425188.1	NCBI	198	27–48	27–48	+	+
*Vigna radiata* var. radiata	XP_014500111.1	NCBI	198	27–48	27–48	+	+
*Juglans regia*	XP_018844524.2	NCBI	205	20–41	28–47	+	+/−
*Triticum turgidum subsp. durum*	VAH03188.1	NCBI	160	7–28	7–28	+	+/−
*Brachypodium arbuscula*	Barbu.8G252800.1.p	Phytozome	153	132–150	129–150	+	−
*Brachypodium* *mexicanum*	Bmexi.01UG208900.1.p	Phytozome	140	108–125	108–125	+	−
*Cucurbita maxima*	XP_022995151.1	NCBI	178	7–28	6–26	+	−
*Cucurbita pepo* subsp. pepo	XP_023545371.1	NCBI	151	132–150	132–150	+	−
*Cryptomeria japonica*	XP_059077992.1	NCBI	144	96–111	96–114	+	−
*Picea abies*	MA_8815334g0010	PlantGenIE	230	203–225	206–225	+	−
*Quercus suber*	KAK7845242.1	NCBI	443	121–141 164–184 221–241	121–146 166–184 221–240	+	−
*Salvia splendens*	XP_042031535.1	NCBI	212	12–29 81–98	12–30 58–75 82–101	+	−
*Gossypium barbadense*	Gobar.D06G228300.1.p	Phytozome	159	137–155	137–155	−	+/−
*Gossypium darwinii*	Godar.A06G226200.1.p	Phytozome	159	138–158	137–157	−	+/−
*Malus domestica*	RXH68939.1	NCBI	569	122–144 152–172 271–291	123–144 150–172 275–295	−	+/−
*Oryza sativa Indica Group*	EEC71462.1	NCBI	328	11–29	12–33	−	+/−
*Carya illinoinensis*	XP_042975722.1	NCBI	128	10–34	22–43	−	−
*Linum usitatissimum*	Lus10029623	Phytozome	100	11–31	12–35	−	−
*Manihot esculenta*	XP_043807697.1	NCBI	162	141–161	141–161	−	−
*Populus trichocarpa*	XP_024465836.1	NCBI	179	26–49	27–49	−	−
*Triticum aestivum*	XP_044417723.1	NCBI	204	43–64	43–67	−	−

Color code: green—there are the active site (+) and the binding motif (+) as in Table 1; light green—the active site is present (+) and the motif contains 1 aa substitution (+/−); yellow—the active site is present (+), but the motif has significantly changed (−); orange—there is no active site (−), but the motif is mostly preserved (+/−); red—both the site (−) and the motif (−) are destroyed.

## Data Availability

The original contributions presented in this study are included in the article/Supplementary Materials. Further inquiries can be directed to the corresponding author.

## Supplementary Materials

The following supporting information can be downloaded at: https://www.mdpi.com/article/10.3390/ijms27010240/s1.

## Abbreviations

The following abbreviations are used in this manuscript:

aa	Amino acid
CK	Cytokinin
ER	Endoplasmic reticulum
HK	Histidine kinase
HPt	Phosphotransmitter
MD	Molecular dynamics
MSP	Multistep phosphorelay
PHP	Pseudo-HPt
RD	Receiver domain
RR	Response regulator
RRB	Type B RR
SE	Standard error
TM	Transmembrane domain (or protein)
TM-HPt	TM-containing phosphotransmitter
