# Supplementary material for: Large-Scale Multigenome-Wide Study Predicts the Existence of Transmembrane Phosphotransfer Proteins in Plant Multistep Phosphorelay Signaling Pathway
Source: Int J Mol Sci. 2025 Dec 25;27(1):240. doi: 10.3390/ijms27010240 (PMC12785465; doi:10.3390/ijms27010240)
Supplement: Supplementary file 1 [file ijms-27-00240-s001.zip › ijms-4043326-supplementary.pdf]

Large-Scale Multigenome-Wide Study Predicts the Existence of Transmembrane Phosphotransfer Proteins in Plant Multistep Phosphorelay Signaling Pathway

Sergey N. Lomin[1], Wolfram G. Brenner[2], Ekaterina M. Savelieva[1], Dmitry V. Arkhipov[1], Georgy A. Romanov[1]

[1]Timiryazev Institute of Plant Physiology, Russian Academy of Sciences, Botanicheskaya 35, 127276 Moscow, Russia;
[2]Universität Leipzig, Institute of Biology, General and Applied Botany, Johannisallee 21-23, 04103 Leipzig, Germany

Supplementary materials

Table S1 (beginning). List of potential TM-HPts and their main characteristics.

Plant species	Protein ID	Database	Protein length (aa)	Predicted TM position: CCTOP	Predicted TM position: Phobius	Active site	Binding motif
Actinidia eriantha	XP_057508184.1	NCBI	179	17-34	20-39	+	+
	XP_057508182.1	NCBI	211	17-34	20-39	+	+
Anthoceros punctatus	Apun_evm.model.utg0000231.837.1	Hornworts	241	198-215	199-219	+	+
Aquilegia coerulea	Aqcoe7G149700.1.p	Phytozome	224	25-52	12-32<br>38-57	+	+
Arachis hypogaea	XP_025611698.1	NCBI	159	14-33<br>52-67	51-68	+	+
	RYR11808.1	NCBI	210	181-201	176-200	+	+
	RYR11810.1	NCBI	174	140-164	140-164	+	+
Brachypodium arbuscula	Barbu.8G252800.1.p	Phytozome	153	132-150	129-150	+	–
Brachypodium mexicanum	Bmexi.01UG208900.1.p	Phytozome	140	108-125	108-125	+	–
Cajanus cajan	KYP37726.1	NCBI	155	139-154	135-154	–	+
Camellia sinensis	XP_028076088.1	NCBI	164	7-32	6-32	+	+
	THG10281.1	NCBI	423	142-157<br>321-336	142-160<br>319-336	+	–
Capsicum annuum	PHT80484.1	NCBI	195	21-40	21-39	+	+
Carya illinoinensis	XP_042975722.1	NCBI	128	10-34	22-43	–	–
Cryptomeria japonica	XP_059077992.1	NCBI	144	96-111	96-114	+	–
Cucumis melo var. makuwa	KAA0051045.1	NCBI	178	155-176	154-176	+	+
Cucurbita argyrosperma subsp. argyrosperma	KAG7036464.1	NCBI	237	11-34<br>41-59	12-34<br>41-63	–	+/–
	KAG7026279.1	NCBI	346	198-216	198-217	+	+
Cucurbita argyrosperma subsp. sororia	KAG6606750.1	NCBI	234	12-34<br>41-59	12-34<br>41-63	–	+/–

Colors: green, there are active site (+) and binding motif (+) as in Table 1; light green, active site (+) and motif with 1 aa substitution (+/-) ; yellow, preserved active site (+) but motif significantly changed (-); orange, no active site (-) but motif is mostly preserved (+/-); red, both site (-) and motif (-) are strongly degraded.

Table S1 (continuation 1). List of potential TM-HPts and their main characteristics.

Plant species	Protein ID	Database	Protein length (aa)	Predicted TM position: CCTOP	Predicted TM position: Phobius	Active site	Binding motif
Cucurbita maxima	XP_022995151.1	NCBI	178	7-28	6-26	+	–
Cucurbita pepo subsp. pepo	XP_023545371.1	NCBI	151	132-150	132-150	+	–
Dendrobium catenatum	PKU83032.1	NCBI	189	136-156<br>166-181	132-155<br>161-181	+	+
Dichanthelium oligosanthes	OEL31897.1	NCBI	167	29-46	29-50	+	+/–
Erythranthe guttata	XP_012858620.1	NCBI	185	164-184	164-184	–	–
	EYU21513.1	NCBI	192	15-36	20-40	+	+
Gastrolobium bilobum	XP_061369588.1	NCBI	351	153-173<br>194-214<br>255-272<br>279-296<br>317-334	153-172<br>192-213<br>252-272<br>278-296<br>317-338	+	–
Glycine max	KAH1225269.1	NCBI	256	188-203<br>230-245	20-38<br>188-207<br>227-245	–	–
	KAH1225268.1	NCBI	256	188-203<br>230-245	20-38<br>188-207<br>227-245	–	–
	KAH1159266.1	NCBI	196	20-41	20-38	–	–
Glycine soja	RZB63908.1	NCBI	206	90-109	90-112	+	+
	RZB63907.1	NCBI	247	57-72<br>131-150	131-154	+	+
	RZC25100.1	NCBI	148	127-146	127-145	+	+/–
	RZB48056.1	NCBI	174	96-112<br>130-146<br>152-173	151-172	+	–
Gnetum montanum	TnS000090365t02	PlantGenIE	193	55-74<br>81-100	55-74<br>80-103	+	+/–

Colors: green, there are active site (+) and binding motif (+) as in Table 1; light green, active site (+) and motif with 1 aa substitution (+/-); yellow, preserved active site (+) but motif significantly changed (-); orange, no active site (-) but motif is mostly preserved (+/-); red, both site (-) and motif (-) are strongly degraded.

Table S1 (continuation 2). List of potential TM-HPts and their main characteristics.

Plant species	Protein ID	Database	Protein length (aa)	Predicted TM position: CCTOP	Predicted TM position: Phobius	Active site	Binding motif
Gossypium armourianum	MBA0839873.1	NCBI	213	191-209	191-209	–	+/–
Gossypium barbadense	Gobar.D06G228300.1.p	Phytozome	159	137-155	137-155	–	+/–
Gossypium darwinii	Godar.A06G226200.1.p	Phytozome	159	138-158	137-157	–	+/–
Gossypium gossypioides	MBA0748386.1	NCBI	205	185-203	185-203	–	+/–
Gossypium hirsutum	XP_040957339.1	NCBI	227	53-72	53-76	+	+
Gossypium raimondii	KJB72520.1	NCBI	158	17-34<br>44-66	18-40<br>46-67	+	+
	KJB72519.1	NCBI	137	15-36<br>46-66	18-40<br>46-67	+	–
Gossypium trilobum	MBA0778665.1	NCBI	207	189-206	189-206	–	–
Helianthus annuus	KAJ0465053.1	NCBI	191	9-27<br>30-49	9-27<br>33-49	–	–
Hevea brasiliensis	XP_021683037.2	NCBI	196	20-40	20-42	+	+
Hibiscus syriacus	KAE8670382.1	NCBI	195	37-57	37-57	+	+
	XP_039066368.1	NCBI	138	22-45	20-46	–	–
Juglans regia	XP_018844524.2	NCBI	205	20-41	28-47	+	+/–
Linum usitatissimum	Lus10029623	Phytozome	100	11-31	12-35	–	–
Lupinus albus	KAE9594631.1	NCBI	154	9-27	45-64	+	+
Malus domestica	XP_028945193.1	NCBI	205	43-64	43-68	+	–
	RXH68939.1	NCBI	569	122-144<br>152-172<br>271-291	123-144<br>150-172<br>275-295	–	+/–
Manihot esculenta	XP_043807697.1	NCBI	162	141-161	141-161	–	–
Musa troglodytarum	URE21152.1	NCBI	221	38-61	39-61	–	–

Colors: green, there are active site (+) and binding motif (+) as in Table 1; light green, active site (+) and motif with 1 aa substitution (+/-); yellow, preserved active site (+) but motif significantly changed (-); orange, no active site (-) but motif is mostly preserved (+/-); red, both site (-) and motif (-) are strongly degraded.

Table S1 (continuation 3). List of potential TM-HPts and their main characteristics.

Plant species	Protein ID	Database	Protein length (aa)	Predicted TM position: CCTOP	Predicted TM position: Phobius	Active site	Binding motif
Olea europaea subsp. europaea	CAA2940084.1	NCBI	177	73-95	69-87<br>93-110	+	–
	CAA3000306.1	NCBI	195	69-96	57-76<br>82-99	+	+
	CAA3000309.1	NCBI	147	69-96	57-76<br>82-99	+	+
	CAA3000307.1	NCBI	177	69-96	57-76<br>82-99	+	+
Olea europaea var. sylvestris	XP_022868488.1	NCBI	201	74-95	69-87<br>93-110	+	+
Oryza brachyantha	XP_040380539.1	NCBI	156	6-24	6-28	+	+
Oryza meyeriana var. granulata	KAF0932251.1	NCBI	159	11-29	7-31	–	+/–
Oryza sativa Indica Group	EEC71462.1	NCBI	328	11-29	12-33	–	+/–
Physcomitrium patens	XP_024369173.1	NCBI	425	77-101<br>161-176	37-55<br>76-98<br>159-176	+	+
Picea abies	MA_8815334g0010	PlantGenIE	230	203-225	206-225	+	–
Populus alba x Populus x berolinensis	KAJ6871759.1	NCBI	237	148-168	148-168	–	–
Populus trichocarpa	XP_024465836.1	NCBI	179	26-49	27-49	–	–
Prunus yedoensis var. nudiflora	PQQ12621.1	NCBI	158	89-112	89-112	–	–
Quercus suber	KAK7845242.1	NCBI	443	121-141<br>164-184<br>221-241	121-146<br>166-184<br>221-240	+	–
Salvia hispanica	XP_047969879.1	NCBI	201	46-63	13-32<br>44-61	+	+
	XP_047969881.1	NCBI	184	46-63	13-32<br>44-61	+	+

Colors: green, there are active site (+) and binding motif (+) as in Table 1; light green, active site (+) and motif with 1 aa substitution (+/-); yellow, preserved active site (+) but motif significantly changed (-); orange, no active site (-) but motif is mostly preserved (+/-); red, both site (-) and motif (-) are strongly degraded.

Table S1 (continuation 4). List of potential TM-HPts and their main characteristics.

Plant species	Protein ID	Database	Protein length (aa)	Predicted TM position: CCTOP	Predicted TM position: Phobius	Active site	Binding motif
Salvia miltiorrhiza	XP_057799853.1	NCBI	171	15-36	12-34	+	+
	XP_057799852.1	NCBI	177	15-36	12-34	+	+
	XP_057799850.1/ XP_057799851.1	NCBI	184	15-34<br>163-179	12-34<br>163-180	+	+
Salvia splendens	XP_042031532.1	NCBI	236	12-29<br>81-98	12-30<br>58-75<br>82-101	+	+
	XP_042031534.1	NCBI	219	12-29<br>81-98	12-30<br>58-75<br>82-101	+	+
	XP_042031535.1	NCBI	212	12-29<br>81-98	12-30<br>58-75<br>82-101	+	–
	XP_042001739.1	NCBI	173	16-32	16-32	+	+/–
	XP_042034917.1	NCBI	165	25-44	26-45	+	+/–
	XP_042034915.1	NCBI	182	24-44	26-45	+	+/–
	XP_042035771.1	NCBI	165	25-44	26-45	+	+
	KAG6389410.1	NCBI	164	13-29	12-29	+	+/–
	KAG6429160.1	NCBI	201	157-177	147-169	+	–
Setariaitalica	XP_022681097.1	NCBI	167	15-34	15-34	–	+/–
	XP_022681096.1	NCBI	167	15-34	15-34	–	+/–
Setaria viridis	XP_034585997.1/XP_034585998.1	NCBI	167	15-34	15-34	–	+/–
Theobroma cacao	XP_017974177.1	NCBI	146	115-142	104-122<br>128-145	–	–
	Thecc.04G025600.1	Phytozome	126	5-28	6-31	–	–
Triticum aestivum	XP_044417723.1	NCBI	204	43-64	43-67	–	–
	XBH59851.1	NCBI	193	43-64	43-67	–	–

Colors: green, there are active site (+) and binding motif (+) as in Table 1; light green, active site (+) and motif with 1 aa substitution (+/-); yellow, preserved active site (+) but motif significantly changed (-); orange, no active site (-) but motif is mostly preserved (+/-); red, both site (-) and motif (-) are strongly degraded.

Table S1 (ending). List of potential TM-HPts and their main characteristics.

Plant species	Protein ID	Database	Protein length (aa)	Predicted TM position:		Active site	Binding motif
				CCTOP	Phobius		
Triticum turgidum subsp. durum	VAH03188.1	NCBI	160	7-28	7-28	+	+/–
Vigna angularis	XP_017425188.1	NCBI	198	27-48	27-48	+	+
Vigna radiata var. radiata	XP_014500111.1	NCBI	198	27-48	27-48	+	+
Vigna umbellata	XP_047160793.1	NCBI	166	15-33<br>56-71	51-70	+	+
	XP_047160792.1	NCBI	170	14-33<br>51-70	51-70	+	+
Yucca filamentosa	YucfiPri.12G151700.1.p	Phytozome	181	69-90	73-94	+	+

Colors: green, there are active site (+) and binding motif (+) as in Table 1; light green, active site (+) and motif with 1 aa substitution (+/-); yellow, preserved active site (+) but motif significantly changed (-); orange, no active site (-) but motif is mostly preserved (+/-); red, both site (-) and motif (-) are strongly degraded.

Table S2. List of plant species with genes encoding only TM-HPts with predicted TM domains.

**Species**	Gene	Transcript	Protein
Aquilegia coerulea	Aqcoe7G149700 (PAC:33072203)	Aqcoe7G149700.1	Aqcoe7G149700.1.p
Brachypodium arbuscula	Barbu.8G252800 (PAC:53286819)	Barbu.8G252800.1	Barbu.8G252800.1.p
Brachypodium mexicanum	Bmexi.01UG208900 (PAC:59920337)	Bmexi.01UG208900.1	Bmexi.01UG208900.1.p
Carya illinoinensis	NC_056755.1	XM_043119788.1	XP_042975722.1
Cryptomeria japonica	NC_081410.1	XM_059222009.1	XP_059077992.1
Cucurbita pepo subsp. pepo	NC_036648.1	XM_023689603.1	XP_023545371.1
Gossypium barbadense	Gobar.D06G228300 (PAC:42288572)	Gobar.D06G228300.1	Gobar.D06G228300.1.p
Gossypium darwinii	Godar.A06G226200 (PAC:42569638)	Godar.A06G226200.1	Godar.A06G226200.1.p
Malus domestica	NC_041799.1	XM_029089360.1	XP_028945193.1
Manihot esculenta	NC_035176.2	XM_043951762.1	XP_043807697.1
Salvia splendens	NC_056050.1	XM_042175601.1	XP_042031532.1
		XM_042175600.1	XP_042031534.1
		XM_042175598.1	XP_042031535.1
	NC_056041.1	XM_042145805.1	XP_042001739.1
Theobroma cacao	NC_030853.1	XM_018118688.1	XP_017974177.1
	Thecc.04G025600 (PAC:42020285)	Thecc.04G025600.1	Thecc.04G273700.1.p
Vigna angularis	NC_068980.1	XM_017569699.2	XP_017425188.1
Vigna radiata var. radiata	NC_028355.1	XM_014644625.2	XP_014500111.1

The protein slots are colored the same as in Table S1.

Table S3 (beginning). Correspondence of protein and CDS/RNA sequences used to generate the phylogenetic tree.

Protein Name	RNA ID	Protein ID	Plant name
**CLADE1 (AHP1)**			
AhTM-HPt1	XM_025755913.2°	XP_025611698.1	*Arachis hypogaea*
LaTM-HPt1	WOCE01000018.1°	KAE9594631.1	*Lupinus albus*
AhTM-HPt2	SDMP01000014.1°	RYR11808.1	*Arachis hypogaea*
GsTM-HPt1	QZWG01000015.1°	RZB63907.1	*Glycine soja cultivar W05*
CsTM-HPt1	XM_028220287.1°	XP_028076088.1	*Camellia sinensis*
GhTM-HPt1	XM_041101405.1°	XP_040957339.1	*Gossypium hirsutum*
HsTM-HPt1	VEPZ02001512.1°	KAE8670382.1	*Hibiscus syriacus* cultivar Baekdansim isolate YM2019G1
GrTM-HPt1	CM001750.1°	KJB72520.1	*Gossypium raimondii*
AHP1	NM_113046.4°	NP_188788.1	*Arabidopsis thaliana*
YfTM-HPt1	YucfiPri.12G151700.1	YucfiPri.12G151700.1	*Yucca filamentosa*
CcTM-HPt1	KQ484075.1°	KYP37726.1	*Cajanus cajan*
OeTM-HPt1	CACTIH010005683.1°	CAA3000306.1	*Olea europaea* subsp. europaea cultivar Farga
OeTM-HPt2	CACTIH010000045.1°	CAA2940084.1	*Olea europaea* subsp. europaea cultivar Farga
OeTM-HPt3	XP_022868488.1°	XM_023012720.1	*Olea europaea* var. sylvestris
CaTM-HPt1	SDJN02000008.1°	KAG7026279.1	*Cucurbita argyrosperma* subsp. argyrosperma cultivar Calabaza pipiana
AHP2	NM_113860.4°	NP_189581.1	*Arabidopsis thaliana*
AHP3(TM)	NM_001344289.1°	NP_001330681.1	*Arabidopsis thaliana*
AHP5	NM_100225.3°	NP_563684.1	*Arabidopsis thaliana*
GsTM-HPt2	QZWG01000002.1°	RZC25100.1	*Glycine soja* cultivar W05
SsTM-HPt1	PNBA02000003.1	KAG6429160.1	*Salvia splendens* isolate huo1
AeTM-HPt1	XM_057652199.1°	XP_057508182.1	*Actinidia eriantha*
CapaTM-HPt1	AYRZ02000006.1°	PHT80484.1	*Capsicum annuum* cultivar CM334
**CLADE2 (AHP6)**			
AHP6	NM_106659.3°	NP_178127.2	*Arabidopsis thaliana*
GgTM-HPt1	JABEZY010000010.1°	MBA0748386.1	*Gossypium gossypioides* isolate 5
GbTM-HPt1	Gobar.D06G228300.1*	Gobar.D06G228300.1	*Gossypium barbadense*
GdTM-HPt1	Godar.A06G226200.1*	Godar.A06G226200.1	*Gossypium darwinii*
**CLADE3 (OsHPt2)**			
OsHPt2	NM_001403691.1°	NP_001390620.1	*Oryza sativa* subsp. Japonica
BmTM-HPt1	Bmexi.01UG208900.1*	Bmexi.01UG208900.1	*Brachypodium mexicanum*
BaTM-HPt1	Barbu.8G252800.1*	Barbu.8G252800.1	*Brachypodium arbuscula*
OsHPt3	NM_001403938.1°	NP_001390867.1	*Oryza sativa* subsp. Japonica
MdTM-HPt1	XM_029089360.1°	XP_028945193.1	*Malus domestica*
JrTM-HPt1	XM_018988978.2°	XP_018844523.2	*Juglans regia*
GmTM-HPt1	JAAFZW020000002.1°	KAH1159266.1	*Glycine max* cultivar EMBRAPA BRS 537
CmTM-HPt1	XM_023139383.1	XP_022995151.1	*Cucurbita maxima*
CupTM-HPt1	XM_023689603.1°	XP_023545371.1	*Cucurbita pepo*

Notations are given as in the figure of the phylogenetic tree. Superscript designations are as in °- GeneBank ID and * - Phytozome ID.

Table S3 (ending). Correspondence of protein and CDS/RNA sequences used to generate the phylogenetic tree.

Protein Name	RNA ID	Protein ID	Plant name
**CLADE4 (OsHPt1)**			
OsHPt1	NM_001401618.1°	NP_001388547.1	*Oryza sativa* subsp. Japonica
OsInTM-HPt1	CM000126.1°	EEC71462.1	*Oryza sativa* subsp. Indica
OsHPt5	NM_001420742.1°	NP_001407671.1	*Oryza sativa* subsp. Japonica
SiTM-HPt1	XM_022825362.1°	XP_022681097.1	*Setaria italica*
SvTM-HPt1	XM_034730106.1°	XP_034585997.1	*Setaria viridis*
OsHPt4	NM_001420232.1°	NP_001407161.1	*Oryza sativa* subsp. Japonica
ObTM-HPt1	XM_040524605.1°	XP_040380539.1	*Oryza brachyantha*
DoTM-HPt1	LWDX02022562.1°	OEL31897.1	*Dichanthelium oligosanthes* cultivar Kellogg
TtTM-HPt1	LT934111.1°	VAH03188.1	*Triticum turgidum* subsp. Durum
OmTM-HPt1	SPHZ02000001.1°	KAF0932251.1	*Oryza meyeriana*
DcTM-HPt1	KZ502144.1°	PKU83032.1	*Dendrobium catenatum*
AcTM-HPt1	Aqcoe7G149700.1*	Aqcoe7G149700.1	*Aquilegia coerulea*
**CLADE5 (AHP4)**			
AHP4	NM_001202983.2°	NP_001189912.1	*Arabidopsis thaliana*
CaTM-HPt1	SDJN02000001.1°	KAG7036464.1	*Cucurbita argyrosperma* subsp. argyrosperma cultivar Calabaza pipiana
Ca(sor)TM-HPt1	JAGKQH010000001.1°	KAG6606750.1	*Cucurbita argyrosperma* subsp. sororia isolate JBR-2021
CmTM-HPt1	SSTE01011420.1°	KAA0051045.1	*Cucumis melo* var. makuwa cultivar SW 3
HbTM-HPt1	XM_021827345.2°	XP_021683037.2	*Hevea brasiliensis*
VuTM-HPt1	XM_047304836.1°	XP_047160792.1	*Vigna umbellate*
VrTM-HPt1	XM_014644625.2°	XP_014500111.1	*Vigna radiata*
VaTM-HPt1	XM_017569699.2°	XP_017425188.1	*Vigna angularis*
Eg-TM-HPt1	KI632223.1°	EYU21513.1	*Mimulus guttatus* cultivar IM62/ *Erythranthe guttata*
**CLADE6 (Salvia TM-HPts)**			
SsTM-HPt2	XM_042145805.1°	XP_042001739.1	*Salvia splendens*
SsTM-HPt3	XM_042175598.1°	XP_042031532.1	*Salvia splendens*
ShTM-HPt1	XM_048113922.1°	XP_047969879.1	*Salvia hispanica*
SsTM-HPt4	XM_042178981.1°	XP_042034915.1	*Salvia splendens*
SsTM-HPt5	XM_042179837.1°	XP_042035771.1	*Salvia splendens*
SsTM-HPt6	PNBA02000020.1°	KAG6389410.1	*Salvia splendens* isolate huo1
SmTM-HPt1	XM_057943867.1°	XP_057799850.1	*Salvia miltiorrhiza*

Notations are given as in the figure of the phylogenetic tree. Superscript designations are as in °- GeneBank ID and * - Phytozome ID.

Table S4. RNA-Sequencing datasets used for the transcriptomic approach to determine expression of TM-HPt-encoding transcripts.

Species	Protein	Dataset (GEO ID)	Tissue	Conditions
Aquilegia coerulea	Aqcoe7G149700.1.p	GSE158507	floral meristem	developmental stages
Camellia sinensis	XP_028076088.1	GSE198198	root	potassium availability
Camellia sinensis	THG1028.1	GSE198198	root	potassium availability
Capsicum annuum	PHT80484.1	GSE240948	leaf	ABA, ET, SA, MeJA
Carya illinoinensis	XP_042975722.1	GSE179336	leaf	drought
Cucurbita pepo ssp. pepo	XP_023545371.1	GSE205063	seed coat	hulled vs. hull-less
Erythranthe guttata	XP_012858620.1	GSE280929	leaf, floral bud	ecotypes, developmental stages
Hevea brasiliensis	XP_021683037.2	GSE101568	latex	normal vs. tapping panel dryness
Hibiscus syriacus	KAE8670382.1	GSE99329	different	flower, leaf, root, ovary
Manihot esculenta	XP_043807697.1	GSE141125	leaf	flowering vs. non-flowering, field vs. mountain
Olea europaea	XP_022868488.1	GSE145100	branches	drought
Physcomitrium patens	XP_024369173.1	GSE138467	protonema	temperature (24 °C, 30 °C, 37 °C)
Salvia miltiorrhiza	XP_057799853.1	GSE100970	hairy roots	MeJA, yeast extract

Abbreviations: ABA abscisic acid; ET ethylene; SA salicylic acid; MeJA methyl jasmonate

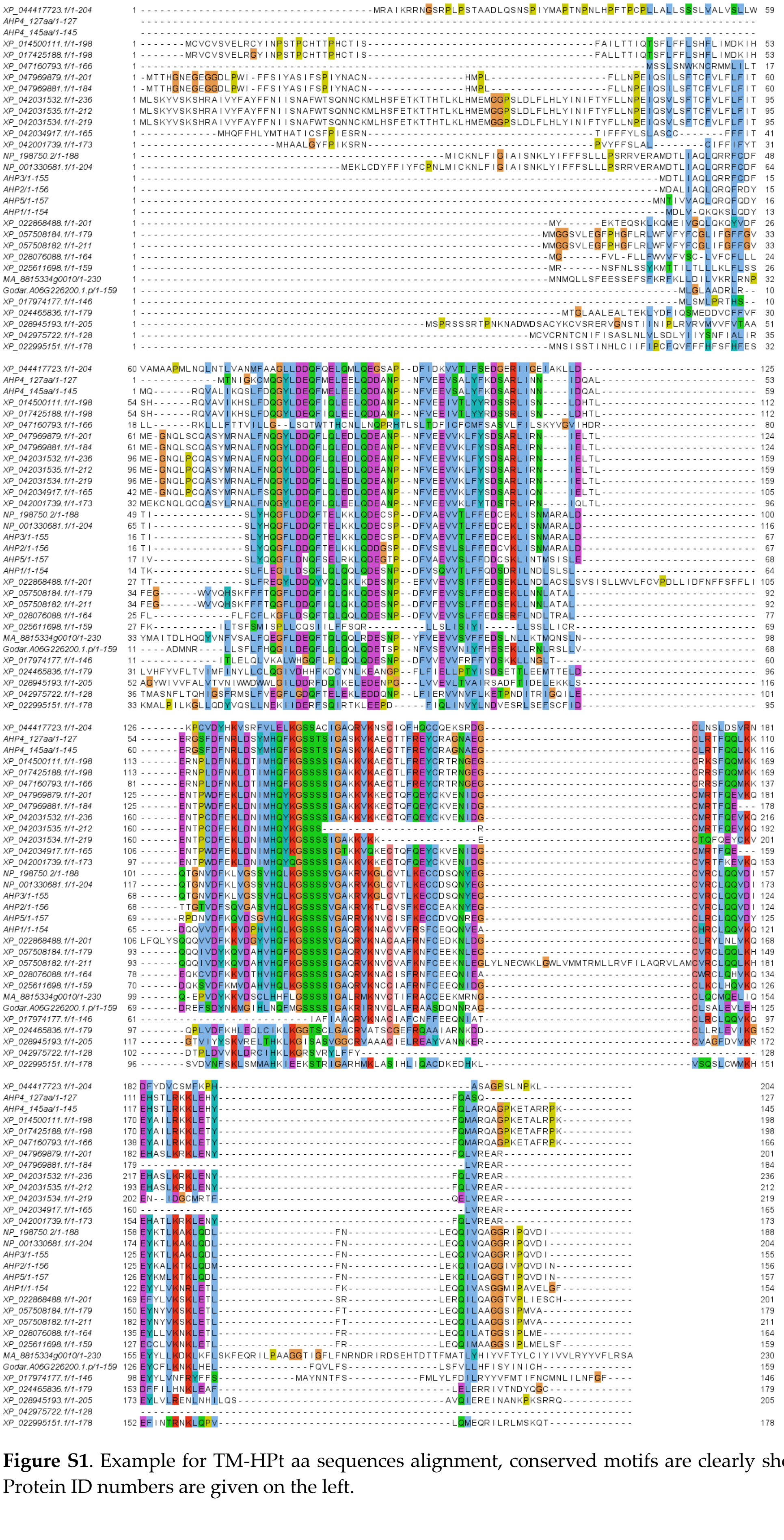

Figure S1. Example for TM-HPt aa sequences alignment, conserved motifs are clearly shown by vertical color bars. Protein ID numbers are given on the left.

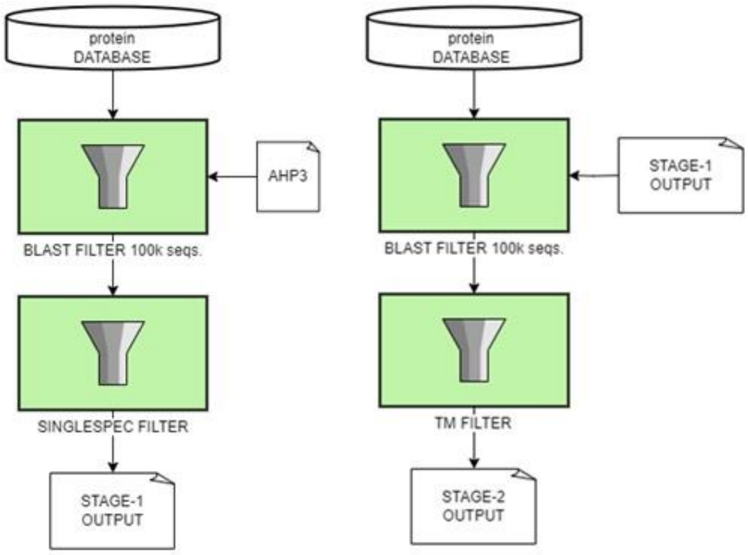

Figure S2. Algorithm of the automatic search for TM-HPts in the NCBI database.

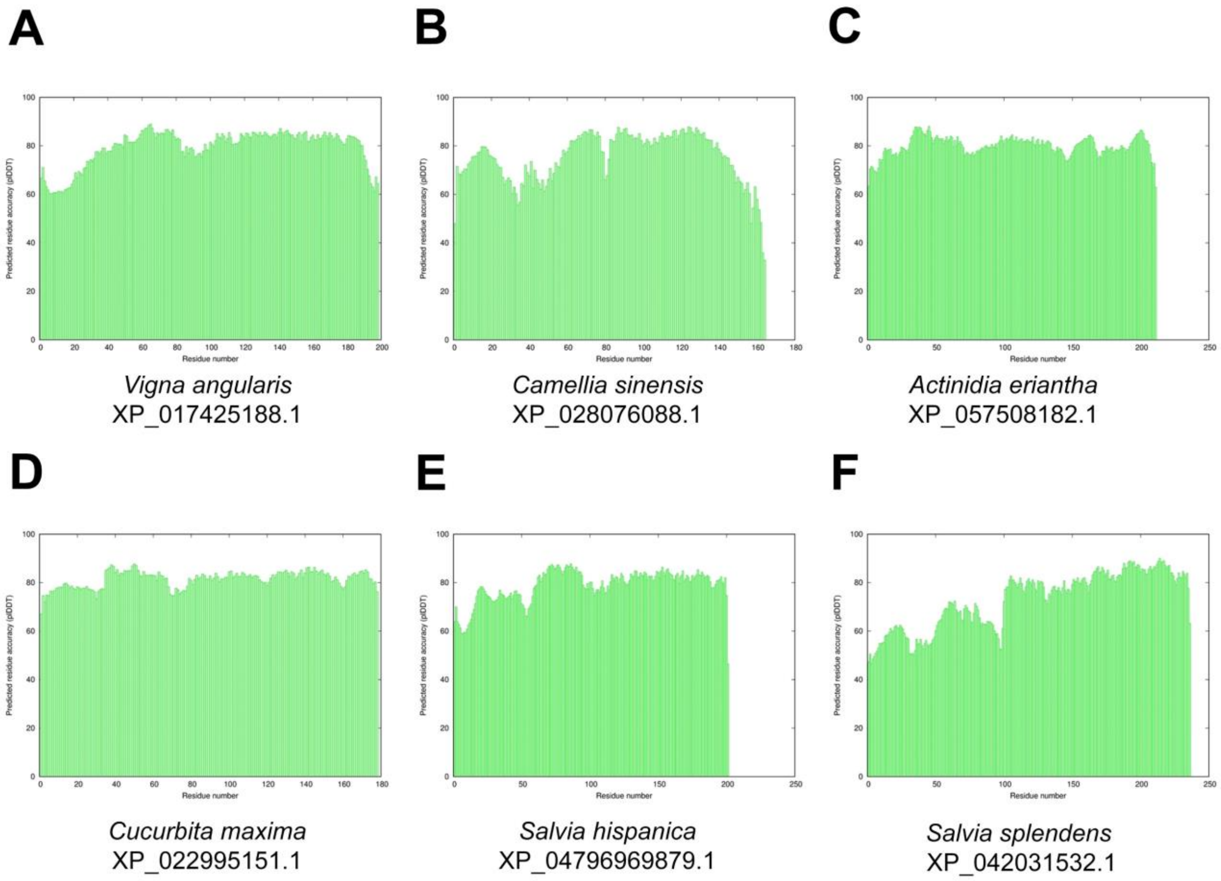

Figure S3. pLDDT values of selected initial models obtained using IntFOLD.
